# Supplementary material for: Optimization of Oligomer Chitosan/Polyvinylpyrrolidone Coating for Enhancing Antibacterial, Hemostatic Effects and Biocompatibility of Nanofibrous Wound Dressing
Source: Polymers (Basel). 2022 Aug 29;14(17):3541. doi: 10.3390/polym14173541 (PMC9460443; doi:10.3390/polym14173541)
Supplement: Supplementary file 1 [file polymers-14-03541-s001.zip › polymers-1840209-supplementary.pdf]

Supplementary Materials for

OPTIMIZATION OF OLIGOMER CHITOSAN/ POLYVINYLPYRROLIDONE COATING FOR ENHANCING ANTIBACTERIAL, HEMOSTATIC EFFECTS AND BIOCOMPATIBILITY OF NANOFIBROUS WOUND DRESSING

Vinh Khanh Doan, Chien Minh Tran, Trinh Thi-Phuong Ho, Linh Kim-Khanh Nguyen, Yen Ngoc Nguyen, Ngan Tuan Tang, Tin Dai Luong, Nhi Ngoc-Thao Dang, Nam Minh-Phuong Tran, Thanh Binh Vu, Hoai Thi-Thu Nguyen, Quyen Thuc Huynh, Hien Quoc Nguyen, Chien Mau Dang, Thang Bach Phan, Hanh Thi-Kieu Ta, Viet Hung Dang, Thanh Dinh Le, Toi Van Vo, Hiep Thi Nguyen*

*Corresponding author: nthiep@hcmiu.edu.vn

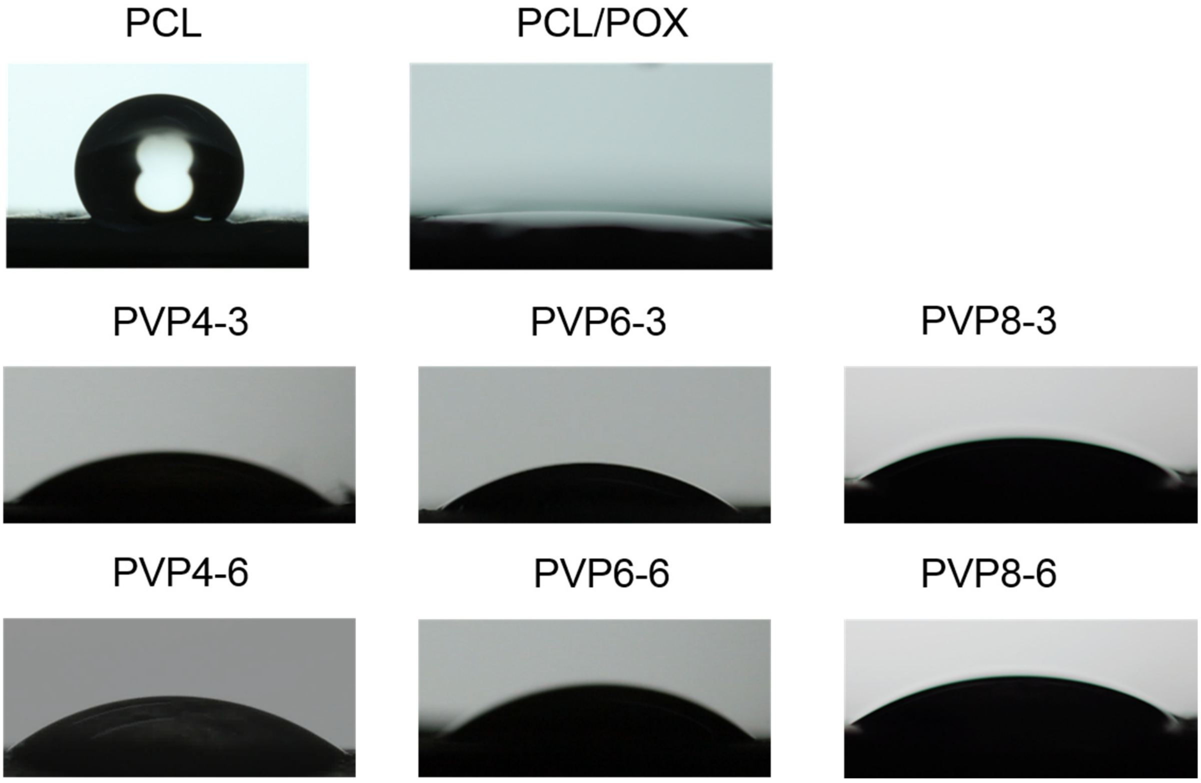

Figure S1. Images of water droplets on PCL, PCL/POX, and PVP/COS surfaces after 0'12”s

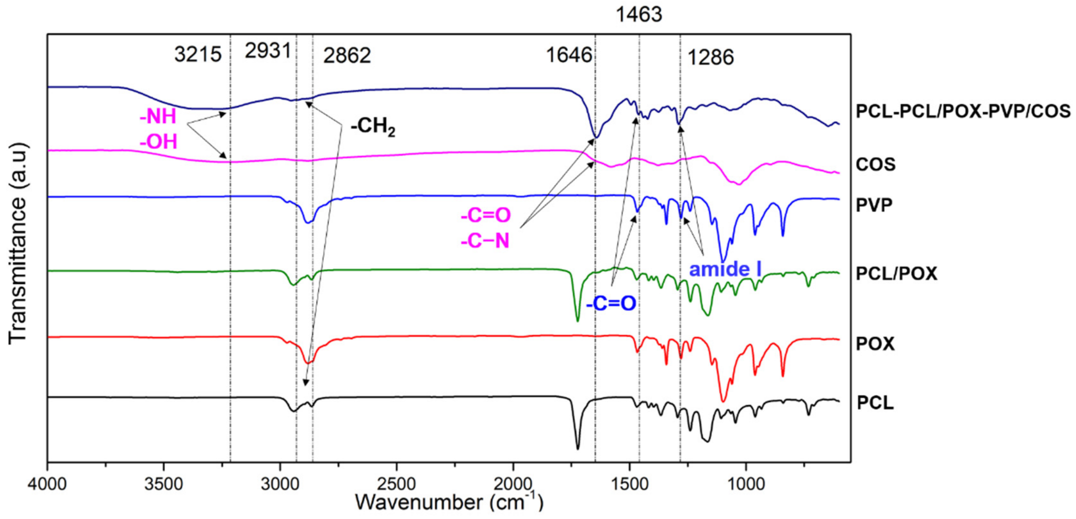

Figure S2. FT-IR spectra of PCL, POX, COS, PVP, and PCL-PCL/POX-PVP/COS membranes

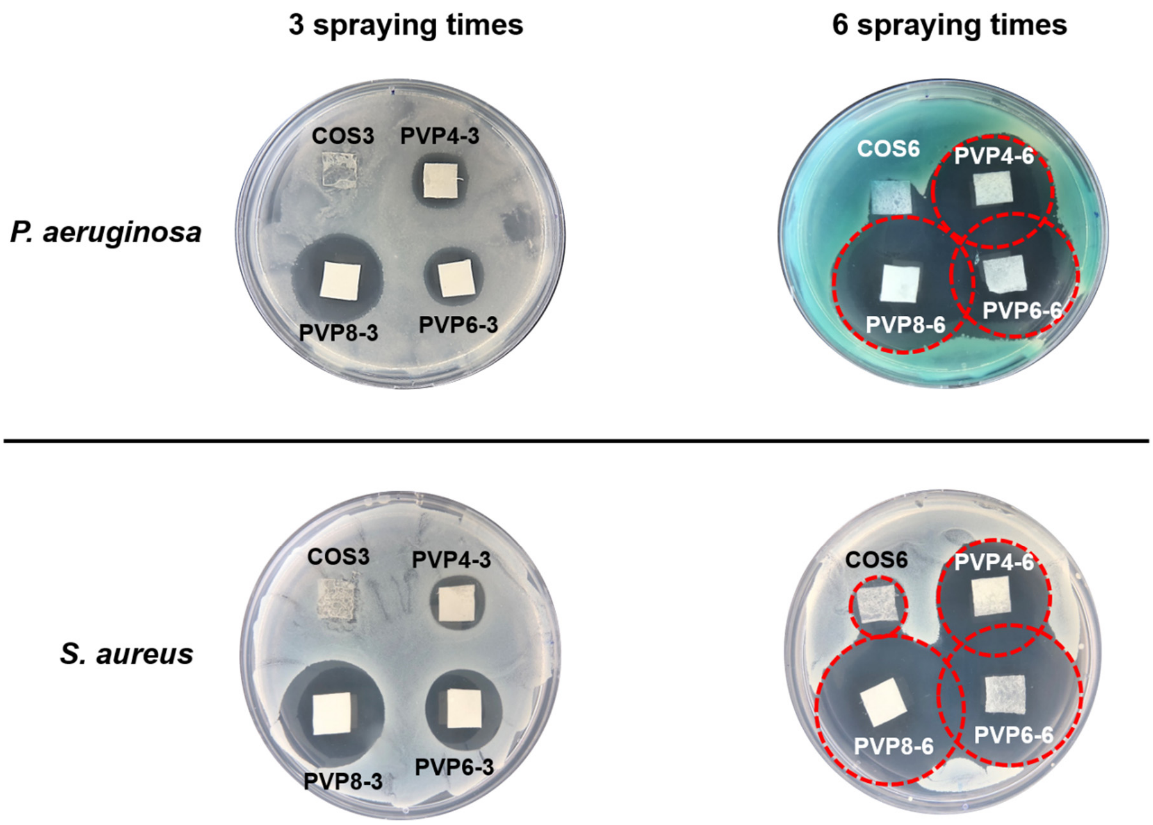

Figure S3: Antibacterial properties of PCL-PCL/POX-PVP/COS membranes with varying PVP concentration and spraying times against *S. aureus* and *P. aeruginosa* by agar disk diffusion test.

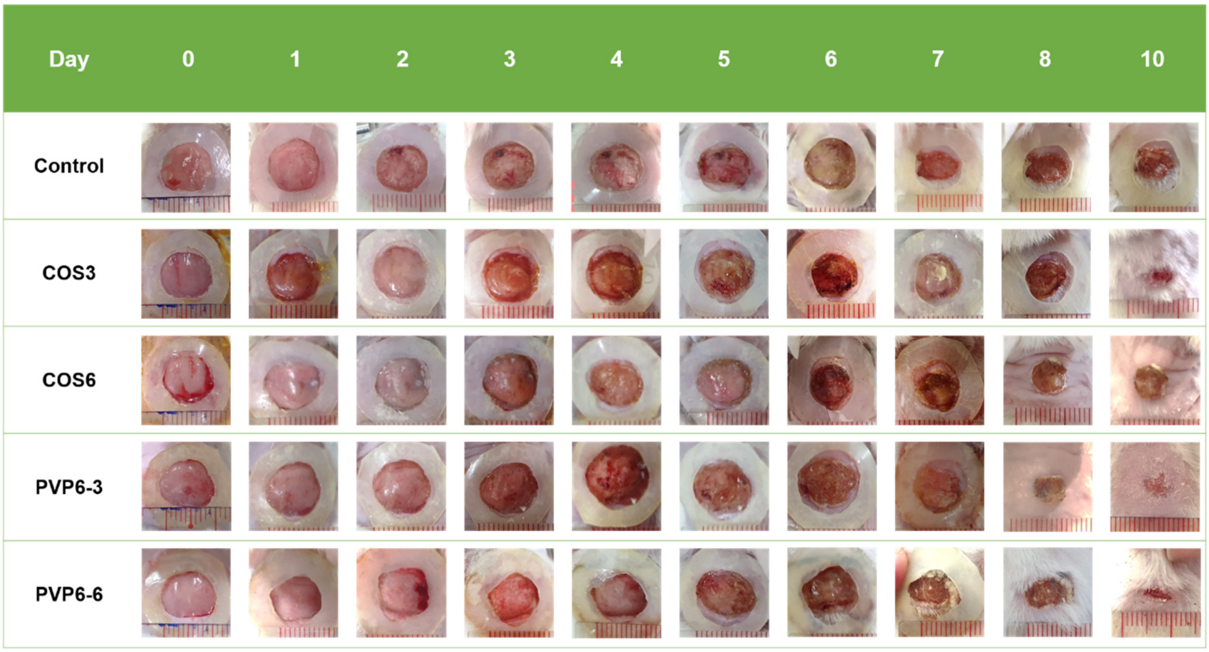

Figure S4. Photographs of surgery sites treated with COS3, COS6, PVP6-3, and PVP6-6 from day 0 to 10.

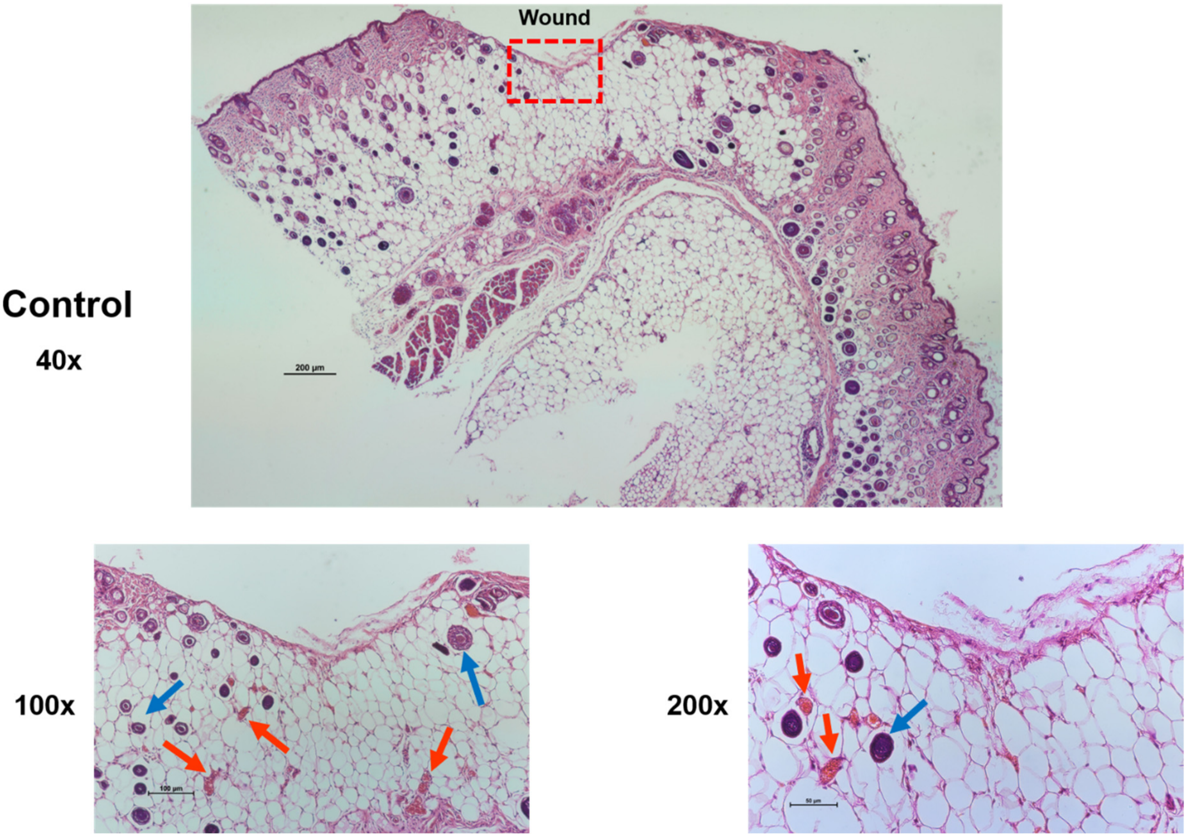

Figure S5. Histological examination of tissues from mice of the control group. Scale bar: 40× – 200 µm, 100× – 100 µm, and 200× – 200 µm. Ep: Epidermis, GT: Granulation tissue. Red arrows indicate blood vessels and erythrocytes. Blue arrows indicate hair follicles. Recovered area is not showed since the wound had not healed.

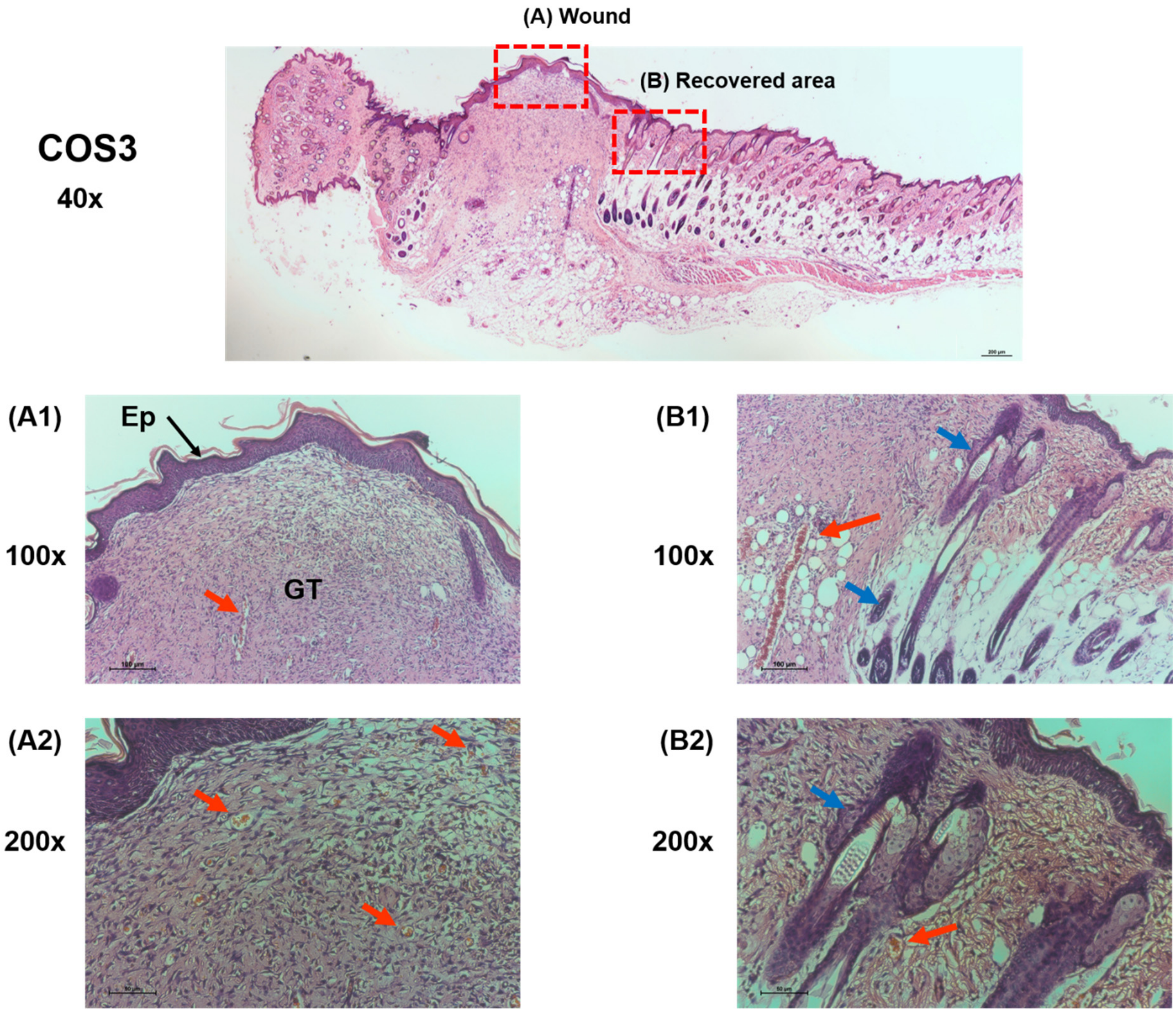

Figure S6. Histological examination of tissues from mice treated with COS3 sample. (A) Wound area (B) Recovered area. Scale bar: 40× – 200 μm, 100× – 100 μm, and 200× – 200 μm. Ep: Epidermis, GT: Granulation tissue. Red arrows indicate blood vessels and erythrocytes. Blue arrows indicate hair follicles.

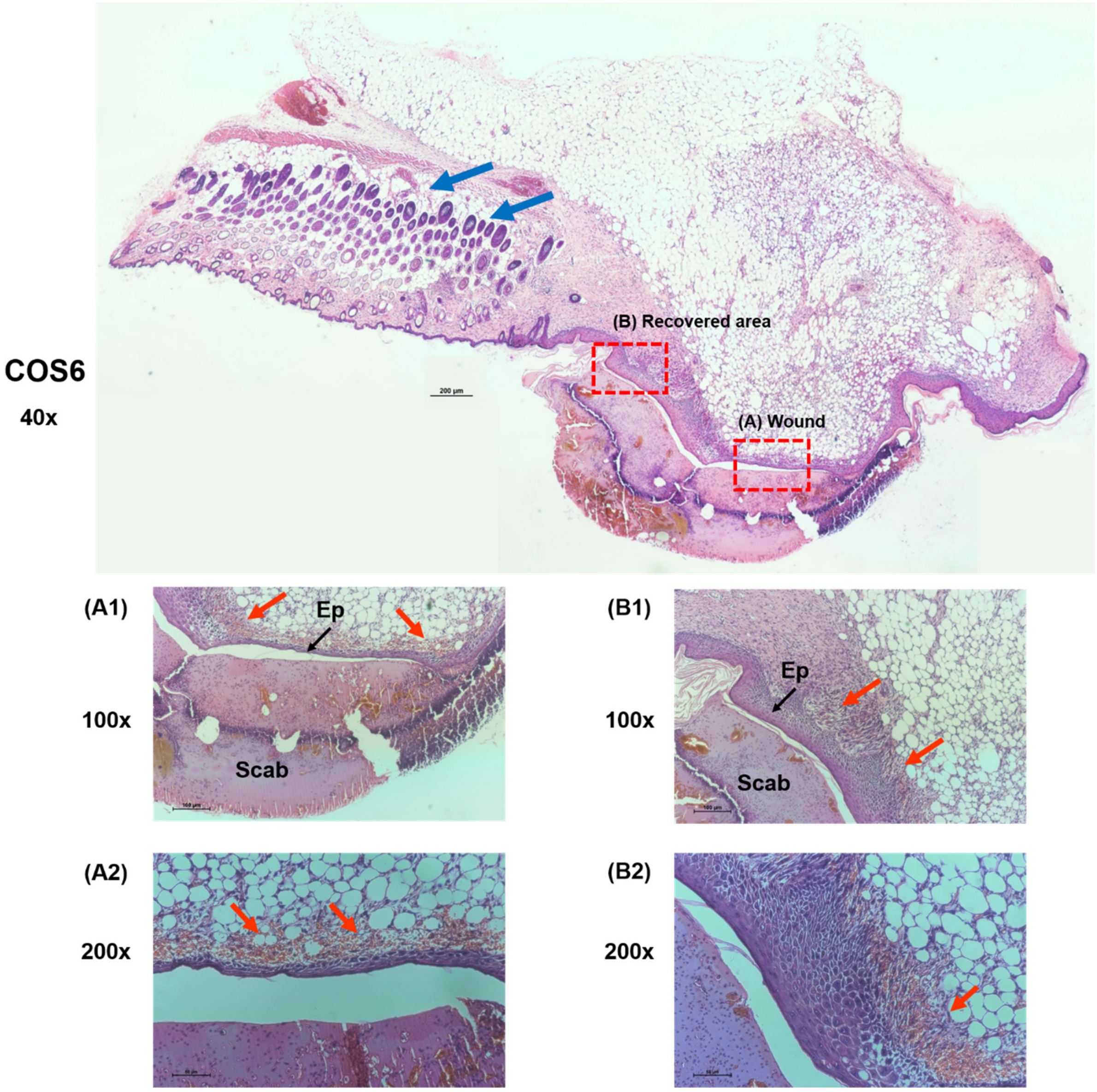

Figure S7. Histological examination of tissues from mice treated with COS6 sample. (A) Wound area (B) Recovered area. Scale bar: 40× – 200 µm, 100× – 100 µm, and 200× – 200 µm. Ep: Epidermis, GT: Granulation tissue. Red arrows indicate blood vessels and erythrocytes. Blue arrows indicate hair follicles.

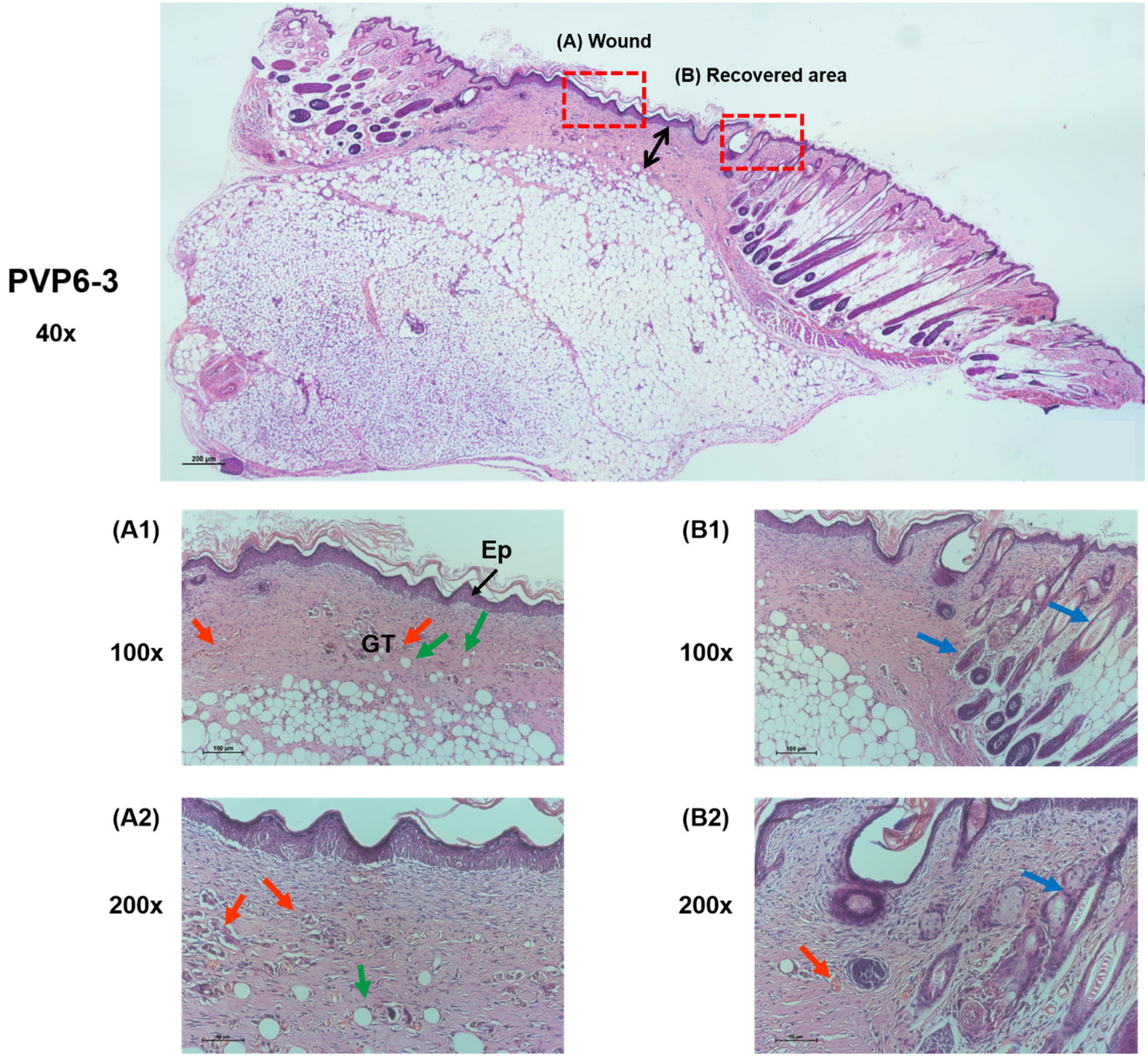

Figure S8. Histological examination of tissues from mice treated with PVP6-3 sample. (A) Wound area (B) Recovered area. Scale bar: 40× – 200 μm, 100× – 100 μm, and 200 – 200 μm. Ep: Epidermis, GT: Granulation tissue. Double-headed arrow indicates a thick dermis layer. Red arrows indicate blood vessels and erythrocytes. Blue arrows indicate hair follicles. Green arrows indicate adipocytes.

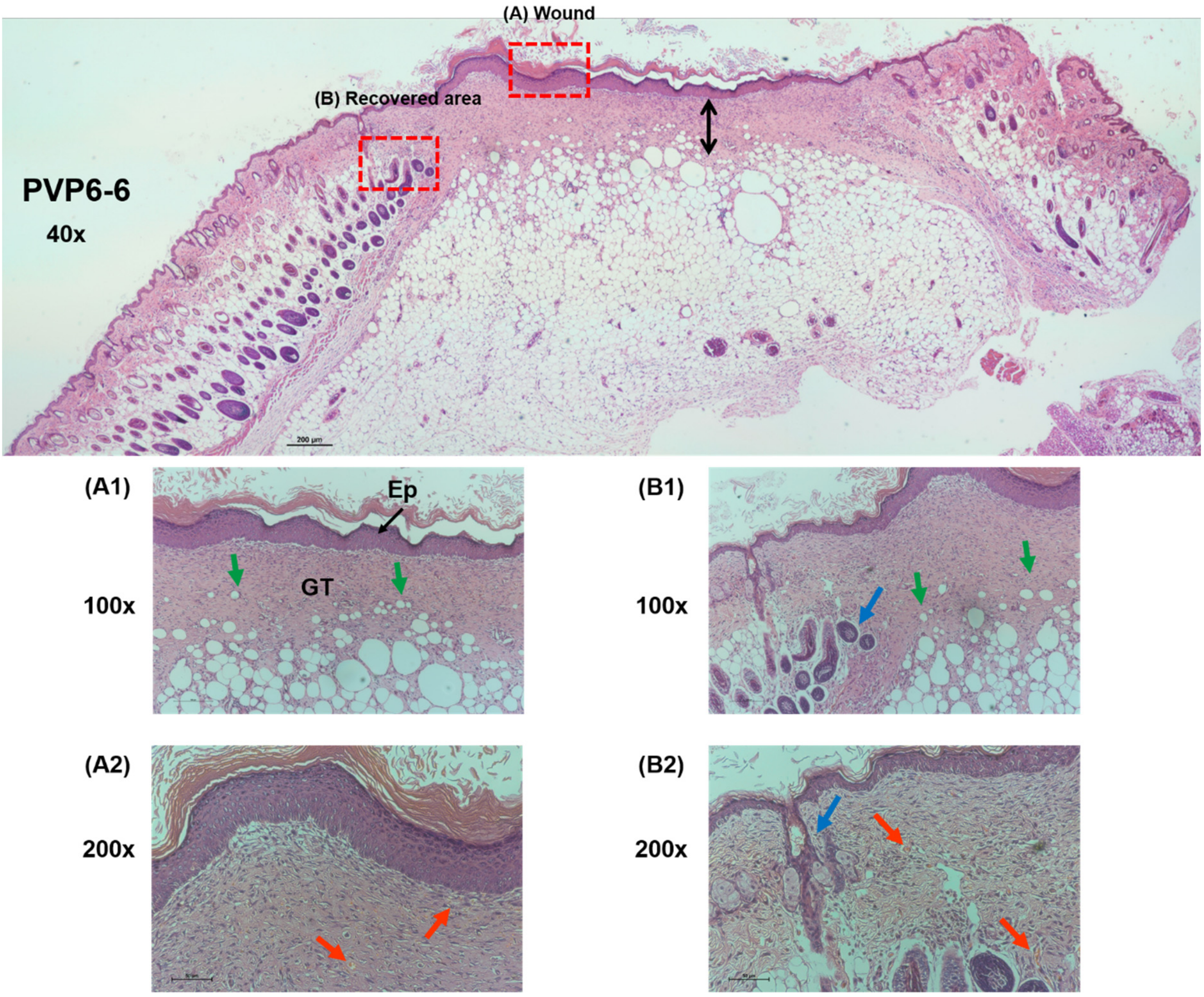

Figure S9: Histological examination of tissues from mice treated with PVP6-6 sample. (A) Wound area (B) Recovered area. Scale bar: 40× – 200 µm, 100× – 100 µm, and 200× – 200 µm. Ep: Epidermis, GT: Granulation tissue. Double-headed arrow indicates a thick dermis layer. Red arrows indicate blood vessels and erythrocytes. Blue arrows indicate hair follicles. Green arrows indicate adipocytes.
